# A Case of Retinal Tear Associated with Use of Sorafenib

**DOI:** 10.3389/fonc.2014.00196

**Published:** 2014-07-24

**Authors:** Kelly M. Gaertner, Stephen H. Caldwell, Osama E. Rahma

**Affiliations:** ^1^Department of Pharmacy Services, University of Virginia Health System, Charlottesville, VA, USA; ^2^Division of Hepatology, Department of Medicine, University of Virginia Health System, Charlottesville, VA, USA; ^3^Division of Hematology/Oncology, Department of Medicine, University of Virginia Health System, Charlottesville, VA, USA

**Keywords:** sorafenib, retinal tear, VEGFR inhibitor, angiogenesis inhibitor, hepatocellular carcinoma

## Abstract

Inhibition of angiogenesis is a target in the treatment of a variety of malignancies. Sorafenib is an oral inhibitor of multiple tyrosine kinases involved in tumor angiogenesis. A related case of ophthalmologic toxicity has been reported with another vascular endothelial growth factor receptor inhibitor, sunitinib. We report the first case of retinal tear possibly associated with sorafenib.

## Introduction

Sorafenib is on oral inhibitor of multiple tyrosine kinases, including vascular endothelial growth factor receptors (VEGFR), approved for the treatment of unresectable hepatocellular carcinoma (HCC) and advanced renal cell carcinoma (1). The most common adverse events noted with sorafenib include hand–foot syndrome, rash, diarrhea, fatigue, and hypertension (2). We report the first case of retinal tear possibly associated with sorafenib.

## Background

A 66-year-old Caucasian male with past medical history remarkable for hypertension was diagnosed with HCC in the setting of non-alcoholic fatty liver disease and cirrhosis. The patient was deemed not to be a candidate for surgical resection or liver transplant given the presence of satellite lesions. His HCC was treated initially with transcatheter arterial chemoembolization (TACE). Subsequently, two months later the patient was found to have disease progression described as multiple new liver lesions. Accordingly, he was initiated on sorafenib 200 mg twice daily that was titrated up by 200 mg weekly until he reached the goal dose of 400 mg twice daily. Three months after being on sorafenib 400 mg twice daily the patient experienced multiple toxicities including dry skin, palm sensitivity, fatigue, nausea, and persistent diarrhea. Therefore, the dose was decreased to 200 mg twice daily, which was better tolerated. After approximately eleven months on the lower dose of 200 mg twice daily, the patient started to complain of blurred vision in the right eye associated with seeing flashes without pain or irritation. The patient’s ocular history was significant for mild myopia and hypertensive retinopathy in both eyes. The patient’s vision was reported to be 20/30 in the right eye and 20/25 in the left eye. The patient’s right eye exam was remarkable for posterior vitreous detachment and three retinal tears while the left eye exam was unremarkable. The patient did not experience any trauma prior to his presentation. The sorafenib was held for two weeks while the patient underwent laser retinopexy. Sorafenib was subsequently resumed at 200 mg twice daily and the patient continued to experience dry skin, fatigue, and palmar sensitivity but no visual changes. Following these events, repeat imaging indicated stability or regression of lesions except slight enlargement of one, which was then treated with TACE. The dosing of sorafenib remained appropriate or below the recommended dose based on hepatic and renal function throughout the course of therapy. No medication changes occurred during this time nor were any drug interactions identified that may have resulted in increased sorafenib exposure.

## Discussion

Many signaling molecules responsible for cancer growth, including VEGF, are expressed in ocular tissues (3, 4). Increases in ocular VEGF are associated with conditions such as proliferative diabetic retinopathy and age-related macular degeneration, which may be treated with intravitreal administration of VEGF inhibitors (3, 5). While the systemic use of VEGF inhibitors is associated with minimal risk of ocular toxicity, there is a known risk of retinal detachment with the use of local, intravitreal therapy (3, 4). In addition, systemic VEGF inhibitors are associated with toxicities that can lead to visual symptoms, such as systemic hypertension, which may result in hypertensive retinopathy (3). We report the first case of possible development of reversible retinal tears on reduced dose sorafenib.

Retinal detachment affects 1 in 10,000 people per year (6). The most common risk factors include aging, myopia, trauma, cataract surgery, and focal retinal atrophy (6). With regards to aging, approximately one in four in the seventh decade of life will develop posterior vitreous detachment, with the incidence increasing in the eighth decade (6). Age and mild myopia are the patient’s only other known risk factors (6). In addition, it is unlikely that the retinal tears in this case were due to hypertensive retinopathy since the patient’s blood pressure was stable throughout treatment with sorafenib.

A similar case has been previously reported with the use of sunitinib in a patient with renal cell carcinoma that was reversible upon discontinuation of sunitinib (7). However, in the sunitinib case the retinal detachment occurred significantly earlier, after only 3 weeks of treatment (7). Sunitinib and sorafenib work by inhibiting multiple intracellular signaling pathways involved in cell proliferation and survival (8, 9). Neither agent purely inhibits vascular targets. Sunitinib has been shown to inhibit VEGFR-1, -2, and -3, platelet-derived growth factor receptors (PDGFR), and c-KIT *in vitro* (10, 11). Sunitinib also produces an active metabolite, SU012662, which has shown equal inhibitory activity toward VEGFR, PDGFR, and c-KIT as sunitinib (10). Sorafenib inhibits VEGFR-2 and -3, PDGFR, c-KIT, RAF-1, and BRAF, which results in inhibition of VEGF receptors as well as downstream activity of VEGF (12–14). Not only does sunitinib have activity at VEGF-1, it also displays more potent inhibition of VEGFR-2 and -3 when compared to sorafenib (8, 13). Reports of retinal complications with systemic use of other VEGFR inhibitors, such as bevacizumab, have not been identified (15).

## Concluding Remarks

This case highlights a potential risk of developing retinal tears while on a systemic VEGFR inhibitor such as sorafenib. Though additional work will be needed to determine causality, this report is of great importance given the increased use of anti-angiogenesis as treatment in a variety of malignancies. Whether the use of other anti-angiogenesis inhibitors could lead to retinal tears remains to be determined and should be reported accordingly. If a patient on a systemic VEGF inhibitor develops visual symptoms, temporary discontinuation of therapy is advised until ophthalmological evaluation can occur (3).

## Author Contributions

All authors contributed to the conception or design, draft or revision, final approval of the version to be published, and agree to be accountable for all aspects of the work.

## Conflict of Interest Statement

Osama E. Rahma is a consultant on the advisory board of Bayer. Kelly M. Gaertner and Stephen H. Caldwell have nothing to disclose.
